# Recent Advances for the Developing of Instant Flavor Peanut Powder: Generation and Challenges

**DOI:** 10.3390/foods11111544

**Published:** 2022-05-24

**Authors:** Yue Liu, Hui Hu, Hongzhi Liu, Qiang Wang

**Affiliations:** Institute of Food Science and Technology, Chinese Academy of Agricultural Sciences, Key Laboratory of Agro-Products Processing, Ministry of Agriculture, Beijing 100193, China; 15552806102@163.com (Y.L.); huhui@caas.cn (H.H.)

**Keywords:** instant flavor peanut powder, heat treatment, flavor, MR, functional properties, peanut meal

## Abstract

Instant flavor peanut powder is a nutritional additive that can be added to foods to impart nutritional value and functional properties. Sensory acceptability is the premise of its development. Flavor is the most critical factor in sensory evaluation. The heat treatment involved in peanut processing is the main way to produce flavor substances and involves chemical reactions: Maillard reaction, caramelization reaction, and lipid oxidation reaction. Peanut is rich in protein, fat, amino acids, fatty acids, and unsaturated fatty acids, which participate in these reactions as volatile precursors. N-heterocyclic compounds, such as the pyrazine, are considered to be the key odorants of the “baking aroma”. However, heat treatment also affects the functional properties of peanut protein (especially solubility) and changes the nutritional value of the final product. In contrast, functional properties affect the behavior of proteins during processing and storage. Peanut protein modification is the current research hotspot in the field of deep processing of plant protein, which is an effective method to solve the protein denaturation caused by heat treatment. The review briefly describes the characterization and mechanism of peanut flavor during heat treatment combined with solubilization modification technology, proposing the possibility of using peanut meal as material to produce IFPP.

## 1. Introduction

Oil extraction is one of the main uses of peanuts in China [1]. The peanut residue produced after pressing through an oil press is also known as peanut meal [2]. Peanut meal is the main by-product of oil extraction, and about 125,000 tons of peanut meal can be obtained each year [1,3].

The protein content of peanut meal is about 55% along with some lipids, carbohydrates, and other nutrient content, such as vitamins A, B, E, K, triterpenoids, sterols, thiamin, riboflavin, and niacin, making it an ideal source of high-quality protein [4]. Fatty acids, triterpenoids, and sterols have been shown to have potential in the fight against diabetes, Alzheimer’s disease, and cancer [5]. Compared with the common protein powder raw material of soy protein, peanut protein is easier for human digestion and absorption (more than 90%), has higher nutritional potency (about equal to 59%), and has no unpleasant beany taste [6]. Additionally, peanut protein has desirable functional properties, such as mixing ability, viscosity, emulsifying ability, and water- and oil-holding capacity [7,8,9,10,11,12,13]. When added to food, peanut protein plays a decisive role in nutritional, organoleptic, physicochemical, and sensory properties (color, texture, flavor) [14,15,16,17,18]. Instant flavor peanut powder (IFPP) has low processing costs. While extracting peanut oil, we can obtain high-quality peanut protein, which effectively increases the added value of the peanut product [19,20].

**Figure 1:**
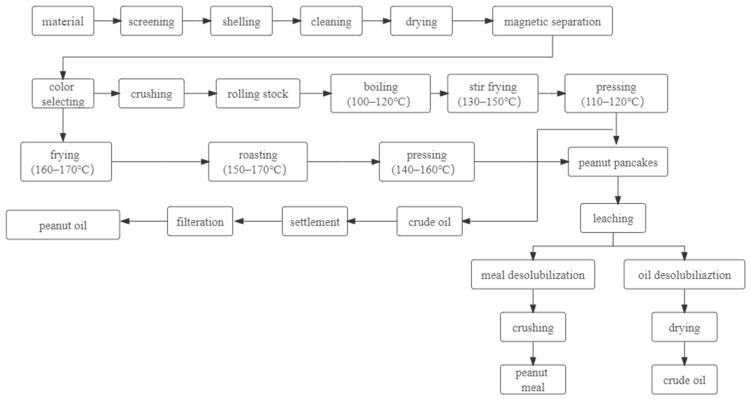
The traditional peanut oil production process.

The heat-treatment technology is an essential part of the peanut industry for the production and manufacture of peanut oil [6], which imparts peanut oil and meal’s distinctive “baking aroma” [21]. The heat treatment of tempering, pressing, crushing, and sterilization in the traditional oil-extraction process can cause a series of complex thermochemical reactions [22], which would cause certain effects on the sensory quality of IFPP. Tempering is an effective means of imparting the typical flavor of peanut oil and IFPP. Roasting, frying, and steaming are effective methods of peanut tempering processes. During the heating process, a series of complex chemical reactions take place, including Maillard reaction (MR), caramelization reaction (CR), lipid oxidation reaction (LOR), and protein thermal degradation [23]. The abundant protein, free amino acids, and unsaturated fatty acids in peanuts become precursors for these reaction. A large number of N, O, and S heterocyclic compounds, such as pyrazine, pyrrole, pyridine, and furan derivatives, and non-heterocyclic substances, such as aldehydes and ketones, are produced during the reaction [24,25], and the interaction between these flavor substances directly affects the sensory evaluation index of the products [20], which is the root cause of the unique “baking aroma” of roasted peanut, peanut oil, peanut meal, and other peanut products. Furthermore, the heat treatments involved in the processing affect the nutritional value and functional properties of the final product [26]. At present, the traditional peanut oil extraction mostly uses a high-temperature pressing process (110–160 °C), which is shown in Figure 1 [27]. While imparting aroma to peanut oil and peanut meal, excessive temperature also affects the spatial structure and degree of polymerization of peanut proteins, resulting in denaturation of peanut proteins, specifically in terms of reduced nutritional value and functional properties (especially solubility) [18], which greatly limits the wide application of peanut proteins in food processing [8,28].

**Figure 2:**
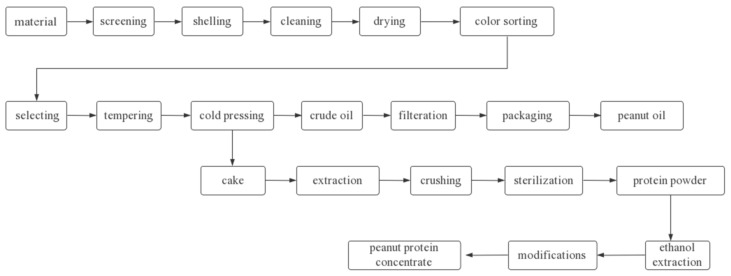
The route of low-temperature peanut oil and protein production.

Solubility is often the first consideration when developing new products and determining the functional properties of a protein component [29]. To solve the problem of severe protein denaturation during the high-temperature pressing of peanut oil, different researchers have invented new pressing and oil-extraction techniques. The focus of their work is concentrated on controlling the heat-treatment temperature during the process. The commonly used preparation methods include cold pressing, leaching, aqueous agent, membrane separation technology, and aqueous enzyme method. These technologies, with the exception of membrane separation, are well-established in the field of oil production. Zhang, Qin, and Sun et al. reviewed the process of making low-denaturing peanut protein powder under different low-temperature conditions, which was effective in reducing the degree of peanut protein denaturation [30,31,32]. In 2004, based on the summary of previous research results, Wang adopted the method of low-temperature cold pressing combined with No. 4 solvent leaching, and through the study of moderate adjustment of peanut oil yield gap and meal thickness [33], established the largest peanut low-temperature pressing oil–protein co-production line in China, and it has reached the international advanced level. The specific process flow is shown in Figure 2.

Although these methods could effectively prevent peanut protein denaturation, basically keeping the protein content and residual oil rate in a suitable range (residual oil ≤ 0.8%, protein ≥ 58%, NSI ≥ 70.6%), the obtained peanut oil and peanut protein powder were significantly worse in aroma than the traditional high-temperature pressing process. This is because the flavor substances produced at low temperatures are limited. With the continuous maturation of protein technology, the modification of denatured peanut protein after heating at the protein molecular level has become possible. Modification is the artificial modification of the structure of a protein, and these modifications are related with delayed deterioration reactions, removal of toxic or inhibitory compounds, as well as incorporation of nutrients and additives through the formation of covalent bonding [34]. At the molecular level, these modifications are made by cutting the main chain of the protein molecule or by modifying the side chain groups of the protein molecule to change the protein structure and conformation at different levels to achieve optimal properties of the protein in terms of size, surface, charge, hydrophobicity/hydrophilicity ratio, and molecular flexibility [15,30]. It is feasible to achieve increased solubility by changing the composition and structure of peanut proteins. According to the different mechanisms of structural modification of proteins, the common means of solubility modification mainly include physical modification, chemical modification, and biological modification [34].

Flavor is an important quality criterion for food [35]. On the basis of ensuring the functional properties of peanut protein, it is imperative to conduct a summary work on peanut flavor and protein modification to reduce the production and economic loss of IFPP caused by flavor deficiency. In order to improve the grim situation of low utilization of peanut meal yield, through the in-depth study of the mechanism of flavor production and microscopic protein structure and function under heating conditions, the use of moderate temperature preheating, medium temperature baking, protein pretreatment to control the degree of peanut protein denaturation, and MR, CR, LOD, and other chemical reactions during the heating process is an ideal production method of IFPP. This paper reviews the research progress on the characterization and generation mechanism of peanut flavor substances during different heat-treatment processes. In addition, it also summarizes the world’s applications related to the solubilization modification technology of peanut protein. This review proposes the feasibility of IFPP product development based on the mechanism of flavor substance generation and solubilization modification methods that combine the sensory and functional properties of peanut protein. Overall, this review can provide more ideas and possibilities for future research on exploring and establishing higher-quality peanut pressed oil production and flavor protein powder co-production lines.

## 2. Characterization of Characteristic Flavor Substances in Raw Peanuts and Heat-Treatment Peanut Products

**Figure 3:**
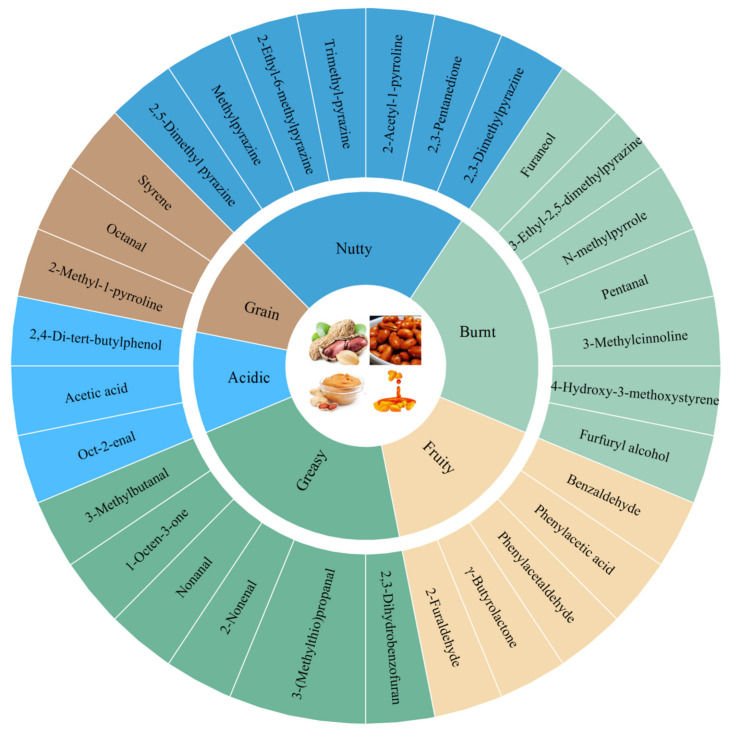
Raw peanuts, roasted peanuts, peanut oil, and peanut butter characteristic aroma components.

Heat treatment is an important step in the production of IFPP, and the various aromatic substances produced during the heating process, such as pyrazine, give it a unique “baking aroma”. IFPP is a new product, and there are no studies in the world to identify and analyze its flavor components. However, flavor characterization of studies for raw peanuts, roasted peanuts, peanut oil, and peanut butter is comprehensive, and IFPP has a similar processing as a by-product of peanut oil production. This paragraph summarizes a review of the research on the flavor characterization content of these four kinds of common peanut products on the market. Although a large number of volatile components have been identified from different processed peanut products, not all of them contribute to the finishing aroma of peanut products. A variety of important aroma substances as well as aroma characteristic components currently analyzed and identified from raw peanuts, roasted peanuts, peanut oil, and peanut butter are summarized in Table 1 and Figure 3. Table 1 summarizes the key flavor substances that have been identified in four different processed peanut products and their aroma descriptions. These substances have a large contribution to the overall aroma attributes of the products even if they present different aroma characteristics, and furthermore, they generally have odor activity values (OAV) ≥ 1 or have a high flavor dilution (FD). On the basis of Table 1, Figure 3 summarizes the common aroma property expressions in the four peanut products and lists the aroma active substances that contribute significantly to them, respectively, which are mostly present in these four peanut products and belong to the characteristic aroma substances of peanut products.

**Table 1:** Key flavor components in peanut heat processed products.

No.	Compounds	CAS No.	Flavor Description	Reported Flavor of Peanut Product	Reference
1	2-isobutyl-3-methoxypyrazine	24683-00-9	Bell pepper-like, earthy	Raw peanuts	[28]
2	Trans-4,5-epoxy-(E)-2-decenal		metallic	Raw peanuts	[28]
3	3-Isopropyl-2-methoxy-pyrazine	25773-40-4	Chocolate, nutty	Raw peanuts	[36]
4	Acetic acid	64-19-7	Sharp pungent	Raw peanuts	[36]
5	2-isopropyl-3-methoxypyrazine	93905-03-4	Earthy, pea-like	Raw peanuts, roasted peanut	[28]
6	Nonanal	124-19-6	Beany aroma	Raw peanuts, roasted peanuts	[36]
7	2-Acetylpyrroline	85213-22-5	Popcorn scent	Roasted peanuts, peanut oil	[36]
8	1-Octen-3-one	4312-99-6	Mushroom aroma	Roasted peanuts, raw peanut	[36,37]
9	Trans-4,5-Epoxy-(E)-2-decanal		Metallic aroma	Roasted peanuts	[23,36]
10	2-Methyl-1-pyrroline	872-32-2	Rice aroma	Roasted peanuts	[37]
11	2-Nonenal	2463-53-8	Greasy	Roasted peanuts	[17]
12	Phenylacetaldehyde	122-78-1	Fruity	Roasted peanuts	[38]
13	Phenylacetic acid	103-82-2	Honey, floral scent	Roasted peanuts	[36,37]
14	Methanethiol	74-93-1	Decomposed aroma	Roasted peanuts	[22]
15	2,3-pentanedione	600-14-6	Nutty	Roasted peanuts	[22,36]
16	3-(methylthio) propanal	3268-49-3	Musty potato, tomato	Roasted peanuts, peanut oil, raw peanut	[23,36,38]
17	3-methylbutanal	590-86-3	Fatty	Roasted peanuts	[37]
18	2-methylbutanal	96-17-3	Musty, cocoa, nutty	Roasted Peanuts, raw peanut	[36]
19	Trans-2,4-nonadienal-trans	6750-03-4	Greasy, fatty	Roasted peanuts	[38]
20	Octanal	124-13-0	Beany	Roasted peanuts, raw peanut	[16,17]
21	Pentanal	110-62-3	Fruity, nutty, berry	Roasted peanuts, raw peanut	[39]
22	N-methylpyrrole	96-54-8	Sweet, woody odor	Roasted peanuts	[38]
23	4,5-dimethyloxazole	20662-83-3	Green, sweet, vegetable	Roasted peanuts	[38]
24	Hexanal	66-25-1	Grassy, refreshing, beany	Roasted peanuts, peanut oil, raw peanut	[23,37]
25	2,3-dimethylpyrazine	5910-89-4	Nutty	Roasted peanuts, peanut oil	[23,38]
26	Oct-2-enal	2363-89-5	Fatty	Peanut oil	[38]
27	Benzaldehyde	100-52-7	Fruity	Roasted peanuts, peanut oil	[23,38]
28	2-Acetyl-3-methylpyrazine	23787-80-6	Rose aroma	Peanut oil	[38]
29	Phenethyl alcohol	60-12-8	Bitter medicinal	Peanut oil	[38]
30	Pyrrole-2-carboxaldehyde	1003-29-8	Sweet fragrance	Peanut oil	[38]
31	Gamma butyrolactone	96-48-0	Alcohol odor	Peanut oil	[38]
32	2-furaldehyde	98-01-1	Green, sweet, and vegetable	Roasted peanuts, peanut oil, peanut butter	[23,39,40]
33	Methylpyrazine	109-08-0	Nutty	Roasted peanuts, peanut oil, Peanut butter	[23,39,40]
34	2,5-dimethyl pyrazine	123-32-0	Nutty	Roasted peanuts, peanut oil, peanut butter	[40]
35	Trimethyl-pyrazine	14667-55-1	Nutty	Roasted peanuts, peanut oil, peanut butter	[40]
36	Furaneol	3658-77-3	Caramel aroma	Roasted peanuts, peanut oil, peanut butter	[36,37]
37	3-Ethyl-2,5-diMethylpyrazine	13360-65-1	Burnt aroma	Peanut oil, peanut butter	[23]
38	4-Hydroxy-3-methoxystyrene	7786-61-0	Burnt aroma	Peanut butter	[40]

In the fact that the aroma attributes of peanut products are the result of many different kinds of flavor substances, the interactions between aroma active substances such as pyrazines, furans, pyridines, aldehydes, ketones, and alcohols are key. Regarding both raw or heat-treatment peanut products, the aroma active substances are the root cause of their different aromas.

### 2.1. Characterization of Raw Peanut Flavor Components

Up to now, about 70 volatile compounds have been found in fresh peanuts, mainly consisting of heterocyclic and non-heterocyclic compounds, of which alkyl pyrazines and furans are the main ones: ethyl 2-ethyl-3,6-dihydroxypropionate (fruity aroma), ethyl 2-methylbutyrate (green apple aroma), ethyl 3-methylbutyrate (fruity aroma), octanal (grassy aroma), hexanal (oily aroma), butyric acid (cheese aroma), isovaleric acid (rotten aroma), pentanoic acid (rotten aroma), isovaleric acid (flower aroma), hexanoic acid (coconut oil aroma), and furfuryl alcohol (burnt aroma), with hexanal and octanal being the main sources of raw peanut bean aroma [22,37,41]. The non-heterocyclic compounds are mainly formed by lipid oxidation and degradation of hydroperoxides [41]. They have a very low aroma threshold, which can only be detected by GC-O [37]. In addition, slight differences in compounds in peanuts are caused by varietal differences, resulting in differences in flavor between peanut species. For example, Argentine peanuts have been described as musty, but Chinese peanuts exhibit a woody/earthy shell/skin flavor as well as bitter and sour flavor, while American peanuts have a sweet, aromatic intensity and are often used in the production of roasted peanuts and peanut butter [42]; Nepote found that high-oleic peanuts contained significantly higher levels of pyrazine than ordinary peanuts and contained lower levels of aldehydes. The flavor of high-oleic peanuts was more stable after prolonged storage [43]. Interestingly, differences in the harvest season of the same peanut also had an effect on peanut flavor. Autumn-ripened roasted peanuts in Taiwan found to contain higher levels of total pyrazine than spring-ripened peanuts, consistent with the general consumer perception that autumn-ripened peanuts have a richer peanut flavor [44]. The overall flavor of raw peanuts is relatively bland, with a low content of aroma active substances but rich in amino acids, sugars, fatty acids, and other flavor precursors. The differences in flavor precursors in raw peanuts directly affect the production of flavor substances during heat treatment. Settaluri found that the carbon skeleton in pyrazine was mainly derived from the degradation of sugar compounds in peanut seeds. Meanwhile, the nitrogen elements in pyrazine, pyrrole, and pyridine are mainly derived from amino acids in peanut seeds [45]; Maga found that some of the pyridines in roasted peanuts were formed from alkanals amino acids. Amino acids are considered to be the most important flavor precursors in peanut seeds [46].

### 2.2. Characterization of Heat-Treatment Peanuts Products

#### 2.2.1. Roasted Peanut Flavor Components

The distinctive flavor of peanuts during roasting is one of the main factors influencing consumer choice and acceptance [47]. The flavor of roasted peanuts has been a hot topic for 50 years, with more than 300 volatile compounds identified. In 1966, Mason and Johnson first proposed that heterocyclic compounds, namely pyrazine, pyrrole, and furan, with low molecular weight produce the typical roasted peanut flavor [48]. Baker also suggested that pyrazines are the main cause of roasted flavor during peanut roasting, with 2,5-dimethylpyrazine being the most relevant to roasted peanut aroma [49]. Pyrazine is the heterocyclic compound containing two nitrogen atoms and has traditionally been considered a key element in the formation of the typical aroma of roasted peanuts. Johnson identified 46 peanut volatile components in roasted peanuts, and most of them were pyrazines [50]. Buckholz and Ho quantified roasted peanut volatiles by a variety of different extraction and isolation methods, and the results of their study indicated that several pyrazine compounds were major contributors to flavor [39]. Methylpyrazine; 2-ethyl-3-methylpyrazine; 2,4,5-trimethylpyrazine; 2,5-ethyl-3-dimethylpyrazine; 2,3-diethyl-5-methylpyrazine; and 3-ethyl-2,5-dimethylpyrazine have been identified as contributing to the roasting odor in roasted peanuts [51]. In addition, 2-acetyl-1-pyrroline, 2-propionyl-1-pyrroline, and 4-hydroxy-2,5-dimethyl-3(2h)-furanone were likewise identified as the main sources of roasting aroma, with 2-acetyl-1-pyrroline, which has a strong popcorn flavor, showing the highest OAV in roasted peanuts [36].

The formation of roasted peanut flavor substances is directly linked to peanut varieties. The importance of variety selection for roasted peanut flavor was discovered long ago. The American Runner-type peanut and the Chinese Four-grain Red were considered ideal for roasted peanut production, with a stronger roasted flavor under the same roasting process. Baker compared four different varieties of peanut by establishing a correlation between pyrazine compound content and roasted peanut flavor [49]. By combining with sensory evaluation scores, he found that peanut genotypes differed in roasted flavor and aroma regardless of roast color. Florida MDR 98 formed the highest levels of pyrazines under the same roasting conditions, followed by Florunner, Georgia Greene, and Sun Oleic, respectively [49]. Nepote roasted 16 different genotypes of normal and high-oleic peanuts at 170 °C for 30 min before subjecting the peanut genotypes to chemical and sensory data by principal component analysis and cluster analysis [43]. It was found that the high-oleic geno-types, 4896-11-C and 9399-10, showed high consumer acceptance in a questionnaire, suggesting that some of the high-oleic peanuts could be substituted for regular peanuts. And as mentioned in 2.1, high oleic peanuts have a higher stability. However, Hu found that the higher initial concentration of characteristic precursors (arginine, tyrosine, lysine, and glucose) in normal-oleic peanuts than in high-oleic peanuts was the underlying cause of the inferior aroma of high-oleic peanut oil compared to normal peanut oil [47]. In addition, the roasting temperature and time parameters had a decisive influence on the formation of flavor substances in peanuts [37].

With advances in flavor omics analysis, it has recently become apparent that pyrazines do not necessarily mean that they play an important role in the flavor of peanuts. If the concentration of the compound is below the sensory threshold, then it makes little contribution to the overall aroma. Surprisingly, Chetschik found that most pyrazines did not have as much impact on the overall aroma of roasted peanuts as expected [22]. Schirack found that thirty-eight compounds were the main contributors to the aroma of roasted peanuts, of which only seven were pyrazines [52]. With the continuous exploration of roasted peanut flavor, it was found that short-chain fatty aldehydes, such as pentanal, hexanal, 2-heptanal, 2-octenal, and phenylethylaldehyde, and the sulfides, such as methyl mercaptan, 2-furan mercaptan, and 2-thiophene mercaptan, were also major contributors to the flavor of roasted peanut [22,36].

In addition, some progress has been made in the study of the off-flavor of roasted peanuts. Basha and Young discovered that the protein fraction capable of producing odoriferous volatiles is a naturally occurring oleic-acid-rich lipoprotein by gel filtration of peanut proteins [53]. The production of N-methylpyrolopentane, acetone, dimethyl sulfide, 2-methyl propanol, pentanal, and hexanal was directly related to the musty, fruity, tongue-burning, and soy flavors of roasted peanuts, suggesting that in addition to lipid oxidation, thermal degradation of proteins may be a source of undesirable flavor compounds in roasted peanuts. In addition, fruity esters, such as 2-methylpropionate ethyl, 2-methylbutyrate ethyl, and 3-methylbutyrate ethyl, as well as high levels of short-chain organic acids, such as butyric, 3-methylbutyric, and hexanoic acids, are associated with the fruity taste of off-flavored fermentation [54]. Ethyl acetate, on the other hand, is considered to be an indicator aroma substance for roasted peanut spoilage. The interaction between different compounds resulted in differences in the aroma of roasted peanuts [55].

#### 2.2.2. Characterization of Roasted Peanut Oil Flavor Components

Peanut oil has a strong nutty and roasted flavor that distinguishes it from other edible vegetable oils [23]. For the production of traditional concentrated peanut oil, crushed peanut seeds are roasted and pressed at around 120 °C, which develops the typical flavor of nuts and roasting. The formation pathways of flavor substances in richly flavored peanut oil are similar to those of roasted peanuts in that all amino acids, lipids, carbohydrates, and other substances in peanuts are subjected to high temperatures, resulting in a series of chemical changes.

Liu and Hu accurately identified flavor substances in peanut oil. 2,3-dimethylpyrazine (nutty); 2,3,5-trimethylpyrazin (nutty); 3-ethyl-2,5-dimethylpyrazine (coffee); 2-acetyl-3-methylpyrazine(roasted); 2-acetylpyrrole (floral aroma); hexanal (grassy); benzaldehyde (fruit aroma); and 3-hydroxy-4,4-dimethyl-γ-butyrolactone (wine aroma) were the main aroma substances in traditional peanut oil, while methylpyrazine; 2,5-dimethylpyrazine; and 2-ethyl-5-methylpyrazine are considered to be the key volatiles contributing to the nutty and roasted flavor of peanut oil [38,47]. Liu suggested that the aldehydes mainly produced in the first and middle stages of seed frying and that the nitrogen heterocyclic compounds produced in the later stages contributed to the flavor of peanut oil. More importantly, 30 min of heating at high temperature (200 °C) is the most important stage in the formation of typical flavor of peanut oil [38].

Compared with traditional high-temperature pressing, cold-pressed peanut oil made from low-temperature conditioning (50–60 °C) and low-temperature pressing (60–70 °C) generally has a poorer flavor [23]. As early as 1972, Brown suggested that 2-heptanal; 2-octenal; 2-nonenal’ and 2,4-dodecenal in low-temperature cold-pressed oil were associated with the greasy and fried flavor of freshly cold-pressed roasted peanut oil [25]. In recent years, Dun identified a total of 101 compounds in hot-pressed peanut oil and only 64 compounds in cold-pressed peanut oil [23]. Aldehydes, ketones, hydrocarbons, alcohols, acids, esters, furans, and pyrroles were detected in both hot- and cold-pressed peanut oil samples, while pyrazines and pyridines were only detected in hot-pressed peanut oil samples. The result confirmed previous experimental results that pyrazines are responsible for the roasted flavor and aroma of peanut oil, with 2,5-dimethylpyrazines showing the highest correlation with roasted peanut flavor and aroma. The volatiles of cold-pressed peanut oil contained high levels of aldehydes, mainly hexanal, nonanal, and (Z)-2-heptenal, accounting for 50.88% to 70.12% of the total volatiles, which was about three times higher than that of hot-pressed peanut oil [23]. This finding is consistent with Hu et al., who suggested that the flavor substances in low-temperature cold-pressed oil are mainly aldehydes [47]. Fatty aldehydes, in particular, produce fatty and grassy odors that bring a significant impact on the overall aroma of peanut oil. Because of the lack of high-temperature roasting, the content of heterocyclic compounds, such as pyrazine and pyrrole, were significantly low, and the peanut oil was low in compounds with roasted and toasted aromas. These results clearly indicate that the difference in volatile flavor substances in peanut oil due to the temperature and duration of high temperature between tumbling and pressing is the underlying cause of the difference in flavor between cold-pressed and hot-pressed peanut oil.

#### 2.2.3. Characterization of Peanut Butter Flavor Components

Peanut butter is a casual food product made from fresh peanut seeds roasted and stripped of their red coating and ground. The flavor profile of peanut butter is similar to that of roasted peanuts, with a strong roasted and nutty aroma, particularly typical of roasted peanuts [40]. Peanut butter flavor formation varies by the peanut plant varieties, picking treatment, roasting processing, and storage conditions factors [56]. The roasting process is the most important measure in the formation of the characteristic aroma of roasted peanuts and peanut butter. The peanut seeds are roasted at high temperatures and undergo a series of chemical reactions, including the MR, CLO, and protein thermal degradation, similar to the aroma presentation mechanism of roasted peanuts. After roasting, the peanuts are ground, and the volatile aroma active ingredients are further emitted, endowing peanut butter with a more intense flavor than roasted peanuts. The MR is generally considered to be the main source of flavoring substances in peanut butter. Heterocyclic compounds, such as pyrazines, furans, and pyrroles, produced by the MR, contribute to the roasted, nutty flavor of peanut butter as important aroma substances. Pyrazines have been considered the most important volatile flavor components in peanut butter [57].

As roasted peanuts are an intermediate product in the peanut butter production chain, the flavor of peanut butter is directly influenced by the volatile substances in roasted peanuts. 2,5-dimethylpyrazine, a characteristic aroma substance of roasted peanuts, was also found by Baker to be an important contributor to the roasted peanut aroma of peanut butter [49]. The same was true for 2-ethylpyrazine; 2,5-diethylpyrazine; 2-ethyl-5-methylpyrazine; and other ethyl-substituted pyrazines. Ethyl substitution of one or more methyl groups can significantly lower the threshold, and these compounds make an important contribution to the roasted flavor and nutty taste of peanut butter [49]. The substitution of pyrazine and the substitution of one or more methyl groups by ethyl groups significantly lowered the threshold, and these compounds contributed significantly to the roasted aroma and nutty taste of peanut butter. The key aroma active substances (OVA ≥ 1) in peanut butter were identified, including 2,5-dimethylpyrazine (grilled, grassy); 2,3,5-trimethylpyrazine (nutty, sweet); 3-ethyl-2,5-methylpyrazine (roasted aroma); and 4-hydroxy-2,5-methylpyrazine (roasted aroma) [40]. Hathorn et al. focused on the phenolics in peanut butter, which generally make roasted peanut wood, hulls, and skins have a bitter odor. Peanut skins resulted in an overall reduction in flavor. The 10% peanut skins resulted in a reduction in overall flavor but were effective in enhancing the antioxidant properties of the peanut butter [58]. Similarly, the flavor of peanut butter is also influenced by the raw material and roasting process, as discussed in detail in Section 2.1.

## 3. Formation of Characteristic Flavor Substances of Heat-Processed Peanuts and the Mechanism of Aroma Presentation

Peanut contains about 50% fat and 25% protein and is rich in monounsaturated fatty acids, free amino acids, and other important flavor precursors [21,23]. These non-volatile substances can be converted into volatile compounds through processing methods, such as heat treatment, fermentation, and storage. In recent years, after research into the formation pathways of flavor substances in processed peanut products, there has been an updated description of the flavor presentation mechanisms of a wide range of processed peanut products. Unfortunately, the reactions involving the formation of typical peanut processed product flavor compounds and their corresponding precursors have not been fully elucidated. This paragraph reviews five reported pathways for the formation of flavor substances in processed peanut products, namely the MR, LOR, CR, and their interactions with each other.

### 3.1. Maillard Reaction

The MR is the most important reaction in the formation of flavor and color in processed peanut products [59]. Also known as the carbamide-ammonia reaction, MR is widespread in food processing, which has been studied worldwide for nearly a century. It was discovered and reported by Louis-Camile Maillard in 1922. The MR is the interaction of reducing sugars and amino compounds, such as amines, amino acids, peptides, and proteins in peanuts. The main products are heterocyclic nitrogen compounds such as furans, thiazoles, thiophenes, oxazoles, pyrroles, imidazoles, pyridines, and pyrazines [21,59]. Long baking and roasting at high temperatures is a necessary prerequisite for the occurrence of MR. Carbon module labeling (comla) technique with multi-reaction kinetics has been successfully used for MR kinetic evaluation and validated MR networks [60].

**Figure 4:**
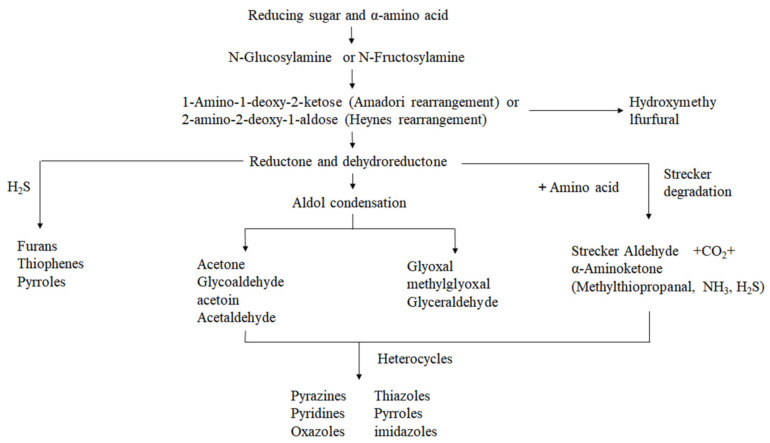
Maillard reaction scheme adapted from Hodge [64].

The generally accepted explanation of the Maillard reaction pathway is that the Maillard reaction is divided into three stages: initial, intermediate, and final [60]. The initial reactions include hydroxylamine condensation to produce cyclized Schiff’s base and rearrangement of the Amodori molecule [61]. The mid-stage reactions mainly include the Strecker reaction, and fructosylamine converts to hydroxymethylfurfural (HMF) or reductone [62]. At the end of the reaction, the intermediates undergo further condensation and polymerization by hydroxyl aldehyde condensation, hydrogen sulfide polymerization, Strecker degradation, and other reactions to finally form N-, O-, and S-containing heterocyclic compounds and the colored pigment melanin (mrps) [63]. Strecker degradation is considered to be the main step in the production of characteristic flavor substances (alkyl pyrazines, aldehydes) in heat-treatment peanut products [32]. MR is very complex, with little known about the details of the reaction process, and its reaction pathway is briefly described in this paper, including the Amadori rearrangement, Heynes rearrangement, aldol condensation, Strecker thermal degradation, and the process of the heterocyclization reaction and the precursors and products in these reaction. The specific reaction pathways are shown in Figure 4 [61].

In order to further elucidate the complex mechanism of the MR in peanuts during heating, Newell tracked the concentrations of precursors and reaction products during different reaction stages of peanut roasting and demonstrated that aspartic acid, glutamic acid, glutamine, asparagine, histidine, and phenylalanine are the main precursors of typical peanut flavors involved in pyrazine formation [65]. Additionally, the role of monosaccharides is very important in the formation of pyrazines. The major soluble sugar component in peanuts is sucrose, followed by glucosamine, hydrosucrose, and raffinose [66]. Mason et al. found that sucrose is involved in the browning reaction by being hydrolyzed to fructose and glucose by converting enzymes during roasting [67].

The reaction products generated by the MR have a characteristic odor profile that essentially affects the quality attributes of the processed peanut product, including flavor, color, and structure [47,60]. For example, pyrazine and thiols were found to be key flavor substances in most cases in roasted peanuts. Buckholz combined sensory evaluation with instrumental analysis of peanuts that had been roasted for various times and found that a decrease in carbonyl and an increase in pyrazine were signs of good peanut flavor, and the change in color was mainly due to the formation of melanin (mrp) [68]. The study demonstrated that one of the carcinogens-acrylamide is associated with the darker color of processed peanut products [59]. The products of the MR have antioxidant effects and also inhibit the oxidation of low-density lipoprotein (LDL) in the human body while reducing cardiovascular and cerebrovascular disease [62]. LDL is the important substrates in lipid-peroxidation reactions that are accelerated in the presence of iron ions and free radicals [69]. Vahid et al. found that oleic acid may reduce the risk of coronary heart disease up to 20–40% mainly via LDL cholesterol content-reducing activity [5].

Although the reaction results in a loss of nutritional quality (destruction of essential amino acids and reduced digestibility), it has beneficial effects on flavor, taste, color, and antioxidant activity.

### 3.2. Lipid Oxidation Reaction

LOR is usually the main cause of “fade” of peanut flavor, resulting in the loss of positive attributes associated with freshly roasted peanuts. The process produces a range of off-flavors, such as “putrid” and “sour”. Pleasant smells, such as “baking aroma” and “sweet aroma”, are masked by these off-flavors [70]. These off-flavors are mainly contributed by aldehydes [54]. Feussner reviewed the two main pathways for the production of aldehydes in peanut oil: thermal oxidation of LOR in crushed peanut seeds and auto-oxidation. The aliphatic aldehydes, alcohols, and ketones formed during heating are mainly produced by auto-oxidation of oils, while some acids, esters, and furans are formed by thermal oxidation of oils and fats [71].

**Figure 5:**
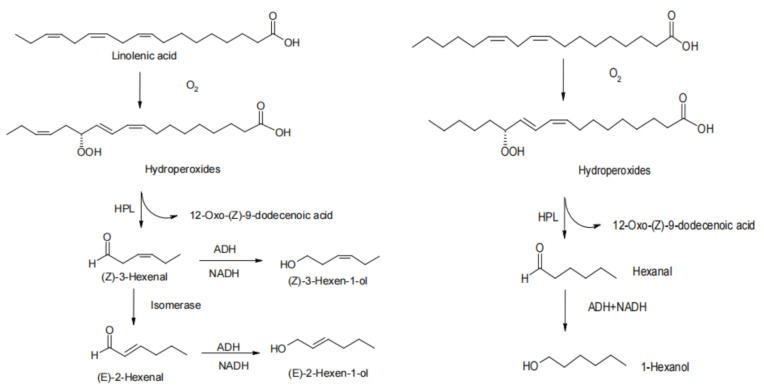
Flavor production process during peanut heating—lipid thermal oxidation reaction [78,79,80].

Peanuts contain a large amount of unsaturated fatty acids (80%) and are highly susceptible to lipid oxidation. Prolonged high-temperature heating creates sufficient conditions for the oxidative degradation of fatty acids to form many iso-olfactory compounds, such as hexanal, heptanal, octanal, 1-octane-3-one, and unsaturated aldehydes, etc. [22]. The resulting mono-hydroperoxides act as the main precursors of iso-olfactory compounds. Of these, hexanal is one of the main oxidation products of linoleic acid esters and has long been used as an indicator of oxidative spoilage of food products. These compounds are directly related to the previously described “paint” and “cardboard” smell of processed peanut products [56]. Flavor “fade” of peanuts due to lipid thermal oxidation is associated with (1) carbonylamine reactions [72]; (2) flavor encapsulation between proteins and lipid hydroperoxides [73]; (3) degradation of heterocyclic nitrogen compounds by lipid radicals and hydroperoxides [74]; and (4) the possibility that the products of lipid thermal oxidation may interact with roasted peanut flavor compounds (pyrazine) and together cause changes in peanut flavor [75]. The reaction pathway for the thermal oxidation of peanut lipids is through auto-oxidation, photo-oxidation, and enzymatic oxidation during the heat process to produce hydroperoxides. Unsaturated fats in peanuts and precursors such as free radicals produce hydroperoxides in the presence of high temperatures and oxygen. The single hydroperoxides continue to oxidize to produce a variety of multiperoxides, which themselves continue to decompose to produce volatile and non-volatile alcohols, aldehydes, ketones, and other substances or undergo polymerization reactions to produce a series of polymers [76,77]. As in Figure 5, the reaction pathway of peanut autoxidation under heating conditions is described: the unsaturated fatty acid linolenic acid in peanuts reacts with oxygen and free radicals under heating conditions to produce hydroperoxides, which are decomposed under the catalytic action of enzymes to produce aldehydes, ketones, alcohols, and other flavor substances.

Furthermore, pyrazines in peanuts are wrapped in free radicals and hydroperoxides from lipid oxidation as thermal oxidation of lipids proceeds, resulting in changes in the flavor of peanut products [75]. The thermal stability of peanut flavor is influenced by peanut variety and water activity. Pattee found that high-oleic peanuts were shown to have better antioxidant properties and better flavor stability than regular peanuts, possibly due to the high production of polyphenolic antioxidants, namely coumaric acid and mrp, during oxidation [81]. Reed investigated the effect of water activity on the flavor stability of roasted peanuts and found that reducing water activity increased the formation of lipid oxidation products and the loss of pyrazine compounds [82]. This finding confirmed the conjecture of Mate et al. that the rate of lipid oxidation in peanuts with low relative humidity (~20%) was higher than that in peanuts with high relative humidity (~60%) [83]. Controlling the water activity in peanuts can effectively reduce peanut lipid thermal oxidation.

However, the effect of peanut lipid thermal oxidation on peanut flavor is not all unfavorable [75]. It is known that most of the non-hybrid compounds in processed peanut products are produced by lipid decomposition, and some aldehydes and ketones provide favorable aromatic odors [70]. For example, phenylacetaldehyde and p-vinyl guaiacol are important contributors to the “fruity aroma” of peanut flour [36]; benzaldehyde and 3-hydroxy-4,4-dimethyl-γ-butyrolactone are key aroma substances for the “floral aroma” flavor of peanut oil. 3-(methylthio) propanal; 3-methylthiopropionaldehyde; and 2,4-decadienal are important for the “frying aroma” of roasted peanuts [23,55]. Alcohols, alkanes, alkenes, and alkynes contribute less to the flavor of peanut heat processing products due to their higher odor thresholds [63]. LOR products make up a smaller proportion of the volatiles of heat-treatment foods and contribute less to flavor than the heterocyclic compounds produced by the MR but play a very important and integral role in the flavor of fats and oils of food [84].

### 3.3. Caramelization Reaction

**Figure 6:**
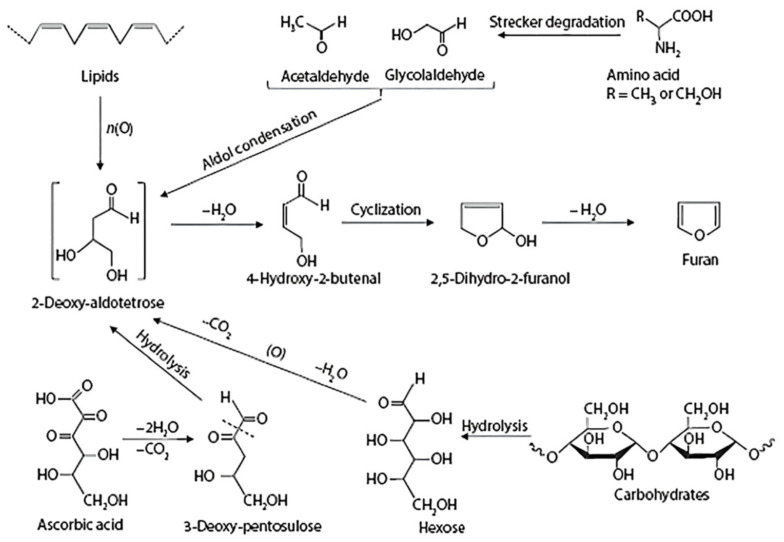
The typical pathway to form furan derivatives adapted from Kroh [86].

CR is an important source of furans and their derivatives in peanut heat-treatment products. Furan derivatives are considered to be the second-largest group of compounds among heat-treatment volatiles, which usually impart caramel-like, sweet, fruity, and nutty flavors to foods [85]. The summary of typical proposed formation pathways of furan from heat-treatment peanuts is shown in Figure 6. Both the CR and the MR are browning reactions, which, at high temperatures (above 140–170 °C), produce a characteristic “baking aroma” flavor and a deepening of the color of the peanut product [86]. The difference with the MR is that the monosaccharides in peanuts are dehydrated and degraded under aerobic conditions by heat without the participation of amino acid compounds [87]. The reaction process includes dehydration, cleavage, and ring formation, resulting in low molecular weight open-chain oxygenates as well as heterocyclic oxygenates, such as furan, furanone, furfural, 5-hydroxymethylfurfural, maltol, and cyclopentenone [88]. The furfural and reduced ketones produced are further involved in the formation of mrp as precursors at the end of the MR [76].

Furfural not only forms the characteristic sweet aroma of caramel in heated foods but is also one of the important precursors for other furan derivatives and an important intermediate for other heterocyclic compounds [23]. Beksan found that hexose can be decomposed into 4-hydroxy-2,5-dimethyl-3(2h)-furanone during heat treatment, of which rhamnose and fructose 1, 6-diphosphate are the key precursors [89]. 4-hydroxy-2,5-dimethyl-3(2h)-furanone has long been recognized as the characteristic flavor substance of peanuts as the characteristic flavoring substance for the “caramel aroma” of peanuts [36]. Two other popcorn-like odor substances, 2-propionyl-1-pyrroline and 2-acetyl-tetrahydropyridine, were first isolated from peanuts and derived from proline and the sugar degradation products hydroxyacetone and 2-oxobutyraldehyde, respectively [54].

## 4. Effect of Heat Treatment on the Quality Characteristics of Peanut Protein

Heat treatment is the most effective way to purify peanut protein and enhance the sensory properties of the product. However, the functional properties of peanut protein in the heating process will change differently with the denaturation and structural changes of the protein, which will accordingly affect a series of indicators of peanut processed products. At the same time, the functional properties can also affect the quality of peanut products. These effects are both positive and negative mainly in the color, taste, flavor, dissolution, and dispersion ability and many other aspects of peanut products [36].

### 4.1. Thermal Degradation of Proteins

Protein makes an important contribution to peanut flavor. On the one hand, it can degrade to form flavor precursors peptides and amino acids [64]; on the other hand, it can interact with flavor compounds to change the head-space concentration of flavor substances and affect the overall flavor of food [54]. The interaction between proteins and flavors is specifically described as the denaturation of proteins by heat during heating; the breaking of hydrogen bonds; the destruction of secondary, tertiary, and quaternary structures of proteins; and the change in spatial structure caused by protein unfolding [90]. Peanut protein and peanut products in part of the flavor substances occurred in a series of reversible and irreversible binding; reversible binding includes ionic interaction, hydrogen bonding, and hydrophobic interactions, while irreversible reactions are mainly the role of covalent bonds, including lysine, carboxyl, disulfide bonds and aldehydes, amines, sulfur-containing compounds, and other flavor substances [47]. As mentioned in Section 2.2.1, thermal degradation of proteins may be an important source of undesirable flavor compounds in roasted peanuts [22]. In the following experiments, Basha conjectured that the protein fractions capable of producing odoriferous volatiles were mainly oleic-acid-rich lipoproteins in peanuts [52], which were thermally degraded and irreversibly covalently bonded to produce N-methylpyrrolopentane, acetone, dimethyl sulfide, 2-methylpropanol, pentanal, and hexanal and associated with musty, tongue-burning, and beany odors in roasted peanuts [35]. Hou identified volatile substances such as nonanal; hexanal; octanal; hexadecanal; octadecanal; 2,3-pentanedione; and 2-acetylfuran in the thermal reaction products of yak meat myogenic fibrin with sugar [91]. Unfortunately, the interaction between protein thermal degradation reactions and food flavor is not well explained. Currently, in the food processing industry, reversible binding of flavor substances through thermal degradation of proteins can be used to reduce flavor loss during processing and to re-release flavor components during consumption, and irreversible binding of covalent bonds for the removal of off-flavors from foods has been a very promising research component.

### 4.2. Effect of Heat Treatment on the Functional Properties of Peanut Proteins

The functional properties of proteins play a decisive role in the nutritional, organoleptic, physicochemical, and sensory properties of peanut products; meanwhile, their functional properties influence the behavior of proteins during processing and storage [92]. Peanut proteins have been shown to have ideal functional properties, which can be incorporated into food products to endue them better nutritional value and organoleptic properties and have been widely used in the food processing industry. However, the reduction of functional properties and nutritional value of the product due to protein denaturation caused by heat treatment seems to be inevitable.

The functional properties of proteins from various oilseed crops have been reviewed by a number of researchers. Koumanov reviewed the relationship between electrostatic interactions of proteins and their functional properties [93]; Schwenke illustrated the relationship between protein structure and functional properties [94]; In the study of the functional properties of peanut proteins, Yamada T. was the first to discover that peanut proteins contain about 10% whey protein and 90% globulins (66% peanut globulins and 23% peanut companion globulins) [95]. Peanut globulins and companion peanut globulins are the major protein fragments in peanut, both of which greatly determine the functional properties of peanut proteins [28]. According to Damodaran, the functional properties of peanut proteins are influenced by a variety of physicochemical properties, such as molecular size, molecular structure, amino acid composition and sequence, net charge, charge distribution, hydrophobicity, hydrophilicity, structure (secondary, tertiary, and quaternary), external environment (PH), etc. [96]. Functional properties of proteins were first explained at the molecular level by Morr et al. The presence and number of charged groups directly affect the solubility and foam stability of proteins due to electrostatic interactions between proteins [97]. Jiao used differential scanning calorimetry (DSC) to determine the thermal properties of the proteins after denaturation: 101.85 °C ± 0.47 °C and 89.68 °C ± 0.28 °C for peanut globulin and peanut companion globulin, respectively [98]. Yang determined the subunit structures of peanut globulin and peanut companion globulin using 2D-PAGE and found that peanut globulin is less soluble, less absorbent, and easily denatured by heat due to the absence of disulfide bonds, which is mainly attributed to the hydrophobic structure of the basic subunit and its heat resistance. Peanut companion globulin is composed of two subunits with different free isoelectric points but the same molecular weight, and the polypeptide composition of different varieties of concomitant arachidonic globulin is basically the same, and its heat resistance is better [99]. Liu found that peanut disulfide protein is mainly composed of polypeptides in the dissociated form but not in the subunit form. It has strong heat resistance that mainly affects the protein’s water absorption, foaming, gelling, and emulsification [100]; in addition, the amphiphilicity of proteins is closely related to the distribution of polar and non-polar residues, interfacial interactions, and foaming and emulsifying activities. The size and composition of amino acids, on the other hand, can influence the sensory evaluation of product flavors by acting on odor receptors [101]. Moure reviewed the effects on the functional properties of oilseed crop proteins during protein processing and classified the functional properties of proteins according to the mechanism of action of the functional properties [28].

However, the functional properties of peanut protein in the process of heat treatment will change differently with the denaturation of protein, which accordingly will affect a series of indicators of peanut processed products. As a result of heat treatment caused by the denaturation of some peanut proteins, denatured proteins show reduced solubility, greatly limiting the use of peanut protein. The solubility of peanut proteins is mainly determined by molecular mass size, amino acid composition, size/composition of subunits, number of disulfide bonds, and hydrophilic/hydrophobic strength [94]. The methods available to assess denaturation of proteins were reviewed by Kilara and Sharkasi [101,102]. Changes in the organization and structure of peanut proteins occur, including structural changes, such as hydrolysis of peptide bonds, breakage of amino acid side chains, disulfide bonds being broken, and condensation of proteins with other molecules [33]. At the same time, the proteins become more compact due to polymerization between them, hydrophilic groups are encapsulated, water solubility is reduced, and they are less susceptible to enzymatic degradation. These variations depend on the intensity and duration of the heat treatment, water activity, pH, salt content, and other active substances [101]. Kinsella found that the solubility of BGPI proteins decreased as the hydrophobicity and sulfhydryl content of the protein surface increased after heat treatment [102]. Han studied the effect of different heat treatments on the solubility of peanut proteins and found that the treatment of roasting at 130 °C for 25 min made the most significant effect on the solubility of peanut proteins [103]. Li found that peanut flour was more difficult to dissolve in acidic beverages after baking than in alkaline conditions [104]. The isoelectric point of denatured peanut proteins ranged from pH 3.5 to 4.5, which is consistent with the experimental results of Chen.

The denaturation of peanut protein is characterized by protein inactivation, reduced water solubility, flocculation and precipitation in water, and reduced nutritional potency. Heat-induced denaturation of peanut proteins, with reduced nutritional value and solubility, limits the widespread use of peanut proteins in food systems. Interestingly, moderate heat treatment can loosen the protein structure and improve solubility. In addition, heat treatment can inactivate anti-nutritional factors, making the protein more easily digested and absorbed by the human body. Studies have shown that the many functional properties of proteins vary in a reciprocal manner; for example, good emulsification and foaming are predicated on higher solubility, while gelation is the opposite [92]. The processing of peanut protein involves physicochemical and thermal treatments that affect the nutritional value and functional properties of the product.

## 5. Research Progress on Solubilization Modification of Peanut Protein

In general, protein solubility is usually required to be above 80% nitrogen solubility index (NSI) for food applications [33]. Unfortunately, most peanut protein products do not exhibit the functional properties required by the food industry, especially solubility, which is attributed to the processing in order to enhance the sensory quality of the product; roasting, frying and other high-temperature treatment; as well as the common organic solvent leaching, high-temperature sterilization treatment, and other process operations. Heat treatment causes aggregation of protein molecules to varying degrees and changes in surface properties, especially the exposure of some hydrophobic groups, resulting in a subsequent decrease in functionality, especially solubility, which severely limits the application of peanut proteins. With the continuous maturation of protein modification techniques, it is possible to modify peanut proteins denatured after heating at the protein molecular level. The common means of solubilization modification mainly include physical modification, chemical modification, and biological modification [105]. As shown in Table 2, a summary of the studies related to the solubilization modification of proteins was conducted in recent years.

**Table 2:** Different forms of protein modification methods.

No.	Authors	Sample	Modification Method	Modification Mechanism	Modified Results	Reference
1	Dong	Pea protein	Cold Plasma Technology	New oxygen- or nitrogen-containing hydrophilic groups are formed on the surface of the protein.	Significant improvement of zein solubility in both neutral and acidic solutions could be observed after treatment with max solubility at 75 V.	[106]
2	Zhao	Peanut protein	Baking	Part of the globulin aggregates or decomposes, improving the solubility of the isolated protein under alkaline conditions.	The solubility at pH 7.0 increased gradually from 76% to 95%.	[107]
3	Zhang	Peanut protein	Microwave	Using microwave effect to change protein aggregation degree and spatial structure.	Under the conditions of microwave power 480 W, modification time 60 s, and pH value 9, the NSI of modified peanut protein concentrate was 53.26%.	[108]
4	Tu	Peanut protein	Dynamic high-pressure microfluidization	As the content of UV-absorbing groups in arachidon increased, the degree of molecular unfolding became larger; as the content of sulfhydryl groups decreased, the three-dimensional structure of arachisin changed locally.	The solubility of arachidrin increased significantly; foaming and foaming stability increased with the increase of homogenization pressure and reached the maximum when the treatment pressure was 120 V.	[109]
5	Li	Egg white protein	Microwave-assisted phosphorylation	The microwave technique can significantly shorten reaction times and accelerate phosphorylation process.	The 3 conditions for optimal phosphorylation modification of egg white are the concentration of sodium tripolyphosphate of 33.84 g/L, microwave power of 419.38 W, and microwave time 90 s for maximum functional properties (solubility, foaming ability, and foaming stability).	[110]
6	Miedzianka	Potato protein	Sodium trimetaphosphate (STMP)	By binding phosphate groups to the active groups of protein side chains, the electronegativity of protein molecules can be changed to increase the electrostatic repulsion between protein molecules and lower their isoelectric points.	The solubility of potato protein increases to 26% at pH 5.2.	[111]
7	Lu	Peanut protein	Sulfonated styrene cation exchange resin	The isoelectric point of the acylate peanut protein shifted, the main protein components are broken into subunits, and the amide group selectively deamidate the protein.	The solubility of modified peanut protein was improved, and the isoelectric point pH was reduced to 0.5–1; the emulsification, emulsion stability, and foaming properties were increased by 215%, 122%, and 538%.	[112]
8	Liu	Peanut protein	Dextran glycosylation	Glycosylation forms protein-polysaccharide complexes by covalent binding of proteins to polysaccharides and the introduction of sugar chains into protein polypeptide chains. Cross-linking of proteins with polysaccharides with hydrophilic hydroxyl groups increases the hydrophilicity of proteins.	Peanut protein nitrogen solubility index increased by 75%.	[113]
9	Qi	Soy protein	Pepsin and phytase complex enzymes	Enzymes modify the amino acid side chain groups of protein molecules by modifying the amino acid side-chain groups of protein molecules to partially degrade or cross-link the protein molecules to polymerize solubility and other functional properties of the protein	The nitrogen solubility index increased from 10.0% to 80.0% at pH 4.0 compared to the unmodified soybean isolate.	[114]
10	Ma	Peanut protein	Limited enzymatic hydrolysis and high-pressure homogenization; compound modification	High-pressure homogenization exposes internal groups of proteins and affects their secondary bonds, increases free sulfhydryl groups in solution, and destroys disulfide bonds, exposing more enzyme cleavage sites, making it easier for enzymes to act on peptide bonds and peptide bonds to break; accelerates protein breakdown.	The nitrogen solubility index of peanut protein concentrate increased to 96.57%.	[34]
11	Zang	Wheat protein	Ultrasonic and Glycosylation Compound Modification	Appropriate ultrasonic treatment is beneficial to the glycosylation modification of wheat gluten, and the surface hydrophobicity of the ultrasonically treated wheat gluten is reduced after grafting with glucose	The solubility of the modified wheat gluten protein is improved in the pH range of 4–7, and the solubility at the isoelectric point is 82.15%.	[115]

### 5.1. Physical Modification

Physical modification of proteins is the use of heating, microwave, ultrasound, high-pressure homogenization, and mechanical action to improve the functional properties of proteins by changing their secondary, tertiary, or quaternary structure and the way protein molecules are aggregated. Mechanical actions such as stirring and crushing have been shown to increase solubility by causing collisions and friction between materials to break up protein particles [116].

Heating, microwave, hygrothermal, and ultrasonic waves destroy the covalent bonds within and between peanut protein molecules through heat treatment, loosening the tightly bound internal structure, opening the hydrogen bonds, and increasing the hydration capacity. Han used hygrothermal treatment of peanut proteins and found that their solubility was increased, and the experimental results were consistent with those of Aminigo [6,117]. Zhao found by SDS-PAGE that with increasing roasting time, the soluble peanut isolate protein content continued to increase under alkaline conditions, and the foaming and emulsification properties first increased and then decreased [107]. Spray cooking has proven to be an effective means of heat-treatment solubilization. Combined with the conclusion of Section 4.2, it seems contradictory that heat treatment can both denature peanut proteins to reduce their solubility and loosen their structure to increase their solubility. A long time ago, Beuchat, Antonio, and many other researchers tried to simulate the industrial heat-treatment conditions of peanut, hoping to establish the relationship between heat-treatment conditions and changes in protein structure [111,118]. Cherry suggested that sustained high temperatures are a prerequisite for the aggregation of proteins into large insoluble aggregates [118]. Adrián showed that within a certain temperature range, the structure of proteins loosens with increasing temperature and solubility increases [119]. However, when this range is exceeded, the molecular motion is intense enough to break the bonds of the primary and secondary structures, and this denaturation leads to aggregation of the protein and thus a decrease in protein solubility. The mechanism of microwave treatment is similar to that of heating treatment. In the microwave field, the protein and water molecules can convert microwave energy into thermal energy, with the protein structure thermally denaturing, the structure loosening, and the solubility increasing. Zhang and Li’s study proved that microwave modification has the advantages of uniform heating, high efficiency, and selectivity over ordinary heat treatment [108,110]; high-pressure homogenization is the most common method for protein solubilization modification. The “cavitation effect” generated by high pressure disrupts the protein peptide chain structure, resulting in a disorganized protein spatial structure and exposing a large number of charged groups leading to increased protein hydration and solubility. Tu used homogenization (0–40 MPa) dynamic microjets to increase solubility of peanut globulin from 3.97 g/L to 7.87 g/L, and the emulsification and foaming properties improved with increasing pressure [109].

Ma found that high-pressure homogenization not only improve the solubility, emulsification, and foaming of peanut protein but also reduce the allergenicity of peanut protein [34]. The mechanistically of homogenization is different from microwave treatment and heat treatment. Non-thermal plasma (NTP) is an emerging area in food research, which also shows that unfolding and exposing hydrophobic sites improve the functional properties of the proteins. Bubler and Dong used NTP to successfully improve the solubility of the soluble protein of corn and pea protein [106,120].

Physical modification has the advantages of simplicity and convenience, short time consumption, and low damage to proteins. However, physical modification mainly changes the advanced structure or intermolecular aggregation of proteins by mechanical action without involving the primary structure of proteins, which has the disadvantages of narrow modification range and limited modification effect. Physical modifications are often combined with chemical or bioenzymatic modifications as a pretreatment for complex modifications.

### 5.2. Chemically Modified

Chemical modification is a method of modifying the protein structure using chemical reagents. The site of action of the modification is the peptide bond, which breaks or reorganizes the internal chemical bonds of the protein or introduces various functional groups. Most studies on chemical modification of arachidonic proteins are based on the derivatization of lysine residues e-amino groups, which directly affect the charge distribution and net charge of protein molecules by introducing various functional groups and selectively derivatizing them with protein side-chain groups, thus affecting the solubility of proteins [121]. Chemical modification can be done by hydrocarbonation (Lys, Cys, Met, His, and Try), oxidation (Cys, Met, His, and Try), acylation (Lys and Tyr), esterification and amination (Glu and Asp), cross-linking, glycosylation, and filling. Acylation, phosphorylation, and glycosylation are the main modalities applied to the chemical solubilization modification of plant-based proteins. Acylation reactions are important for improving the functional properties of proteins, in particular succinylation and acetylation reactions [112]. Meanwhile, peanut and other plant proteins contain a large number of amide groups, and selective deamidation of proteins can improve the functional properties of peanut proteins. The introduction of succinyl groups can weaken the gravitational force between the amino and carbonyl groups in peanut protein molecules. The method change the molecular structure of peanut protein, making the protein molecules tend to stretch from folding, reducing the size of protein aggregates, increasing the net negative charge in peanut protein, moving the isoelectric point to low pH, and finally improving the solubility of peanut protein under acidic conditions significantly. Monteiro found that the main components of peanut protein, namely arachidonin, concomitant arachidonin I, and concomitant arachidonin II, could be modified by succinylation to lower their isoelectric points below 4 and improve their solubility and dispersibility [98]. Lu investigated the effects of succinylation modification factors on the modification effect and structural properties of peanut protein and found that the concentration of peanut protein and succinic anhydride addition were the significant influencing factors through the effect surface analysis [122]. Beuchat further reacted defatted peanut protein powder with different concentrations of succinic anhydride at pH 7.4–8.0. By using polyacrylamide gel electrophoresis, it was found that the main protein fraction broke into subunits [118]. Soluble nitrogen decreased at pH < 4 and increased between pH 6 and 7. Its water-holding capacity, water absorption, and emulsification increased, while viscosity increased significantly. The research work on the modification of peanut protein by succinylation is still in its infancy in this field. The modification of peanut proteins by protein phosphorylation has been shown to be safe and feasible. The mechanism is to use protein side-chain reactive groups to selectively bind phosphate groups to change the electronegativity and intermolecular electrostatic repulsion of protein molecules to make them more easily dispersed in food systems, thus improving the solubility and other functional properties of proteins [123]. Sung found that phosphorylation modification is the introduction of a large number of phosphate groups through the selective use of side chain groups such as hydroxyl. As the protein side chains continue to bind phosphate groups, the electrostatic repulsion and electronegativity between the peanut protein molecules change [124]. Pan, Xiong, and other experimental results proved that protein phosphorylation can effectively improve the solubility, emulsification, foam stability, and water absorption of peanut protein [125,126].

The MR can also be used to covalently bind proteins to polysaccharides (protein-glycan graft modification). By adding sugar compounds, protein polypeptide chains are covalently bound to sugar chains under heating conditions to form protein-polysaccharide complexes [90]. The cross-linking reaction between the protein and the polysaccharide with hydrophilic hydroxyl groups enhances the hydrophilicity of the protein and limits the interaction between the protein molecules, thus contributing to the improvement of the functional properties of the protein [127]. Liu graft-modified peanut isolate protein modified with dextran dry heat treatment for 7 d under heated conditions and showed a 75% increase in NSI of pH 4.0 compared to the peanut isolate protein control [128]. Protein-glycan graft modification resulted in a significant decrease in the α-helix and irregular curl of peanut proteins, a significant increase in the β-fold, and denaturation of proteins, resulting in reduced sensitivity to pH and increased solubility under acidic conditions [129]. In the process of MR, polysaccharides and proteins can also be combined by electrostatic interactions to form soluble complexes, which is conducive to improving the solubility of peanut proteins. Chitosan, because of its positive charge, can be combined with the negatively charged groups of protein relying on electrostatic interactions so that peanut protein has a certain amount of positive charge at the isoelectric point [98]. Yuan and Guo shifted the isoelectric point of peanut isolate by adjusting the protein–chitosan complex ratio, which improved its stability and solubility under acidic conditions [130,131].

Chemical modification can effectively alter the functional properties of proteins, but chemical solvent residues and toxicity of derivatized proteins are difficult to avoid. The protein–glycan graft modification associated with MR is currently the most promising chemical modification modality, but clearly predicting the cross-linking conditions of glycan components with peanut proteins is difficult [113].

### 5.3. Enzymatic Modification

Enzymatic modification is considered to be the most researched and promising way of protein modification [132]. The enzymatic modification has the advantages of high specificity, mild reaction conditions, controllable influencing factors, and good functionality of hydrolysis products [133]. Modification of the side-chain groups of amino acid residues of proteins by selected proteases results in peptides with desirable functional properties. Restriction enzymatic digestion has been widely reported to effectively improve the solubility of peanut proteins. Beucha reported the changes in solubility, water absorption, and emulsification of defatted peanut protein after hydrolysis by pancreatic protease, pepsin, and pineapple protease [118]; Sekul et al. found that papain-treated peanut protein had higher solubility and hydration capacity than the corresponding untreated peanut protein by blank control [134]. Li successfully improved the solubility of concentrated peanut protein in water by using neutral protease [135]. The application of enzymatic modification can solve the limitation of the modification caused by the uniqueness of the enzyme and some studies have shown that the use of alkaline protease and flavorzyme for deep hydrolysis of peanut protein can significantly improve the degree of hydrolysis of peanut protein, and the hydrolyzed protein has better solubility and better properties. It should be noted that excessive enzymatic hydrolysis tends to produce bitter peptides, which adversely affects the sensory quality of peanut protein products [136].

## 6. Conclusions

As people’s consumption levels continue to rise, food with a single nutritional composition can no longer meet consumer demand for health. Theoretically, adding plant protein to traditional food products not only enhances the flavor of the product but also improves the nutritional value of the food, which is currently a hot spot in food development research. As described in Section 1, peanut protein has advantages that other vegetable proteins cannot match, and the use of peanut meal, a by-product of oil extraction, as a raw material for the production of IFPP can not only save costs and increase the added value of peanut meal but also give the food a pleasant, flavorful taste and nutritional value.

The importance of peanut proteins in the human diet is based on the nutritional quality and the functional properties. However, there is not a unique methodology or protocol for leading to optimal products. In fact, food development based on consumer preferences is very important for the production of goods. Even if there are adequate nutritional and physiological functions, consumers will not accept them if their preferred functions do not meet their expectations. Flavor, as an indicator that must be taken into account in the processing of peanut flour, is key to controlling the factors that influence flavor during the milling process, especially as the heat-treatment process involves the MR, CR, and LOR that are primary in the production of flavor substances. The review summarizes the recent investigations on how compounds formed during these reaction influence the sensorial properties (color, flavor, and texture) of heat-treatment peanut products and also summarizes that processing of peanuts affects the functional properties of the proteinic products and their improvement through protein modification, which should be addressed, including physics, chemical, and enzymatic technologies applied to obtain products with desirable properties for food applications.

In summary, heat treatment is a necessary way to develop and produce IFPP. The functional properties of IFPP can be modulated by carefully selecting both original seed, adjustment of heat-treatment time and temperature, and the operational variables during protein modification (pH, temperature, solvent, presence of salts, ionic strength, etc.) to establish the interaction between peanut flavoring mechanism and protein functional properties to achieve the balance between sensory properties and nutritional value of peanut thermally processed products. In conclusion, an effective protein modification approach can balance the content of protein denaturation due to heat treatment to enhance the functional properties of peanut products. Moreover, the study of the interaction between protein thermal degradation and flavor components is the technical key to establish the link between flavor substances and protein functional properties to rationally control the changes of functional properties during heat treatment and to develop IFPP.

## Data Availability

The data presented in this study are available on request from the corresponding author.
